# Scarcity mindset reduces empathic responses to others’ pain: the behavioral and neural evidence

**DOI:** 10.1093/scan/nsad012

**Published:** 2023-03-08

**Authors:** Wanchen Li, Jing Meng, Fang Cui

**Affiliations:** School of Psychology, Shenzhen University, Shenzhen 518060, China; Key Laboratory of Applied Psychology, Chongqing Normal University, Chongqing 401331, China; Research Center for Brain and Cognitive Science, Chongqing Normal University, Chongqing 401331, China; School of Psychology, Shenzhen University, Shenzhen 518060, China; Center for Brain Disorders and Cognitive Neuroscience, Shenzhen University, Shenzhen 518060, China

**Keywords:** scarcity mindset, empathy, event-related potentials, N1, late positive potential

## Abstract

Resource scarcity pervades our life. A scarcity mindset triggered by perceiving insufficient resources has been proven to influence our cognition and behaviors, yet it remains unknown whether this mindset specifically influences empathy. The present study induced feelings of scarcity or abundance in separate groups of participants through experimental manipulation and examined the effects of both mindsets on the behavioral and neural responses to others’ pain. Behaviorally, pain intensity ratings of others’ pain were lower in the scarcity group than in the abundance group. The analysis of event-related potentials revealed that N1 amplitudes for painful and nonpainful stimuli were comparable in the scarcity group but differed significantly in the abundance group. Additionally, while both groups showed larger late positive potential amplitudes for painful stimuli than for nonpainful stimuli, this amplitude differential was significantly smaller in the scarcity group than in the abundance group. Thus, behavioral and neural evidence suggests that inducing a scarcity mindset significantly dampens the ability to empathize with others’ pain during both the early and late stages of empathic processing. These findings shed light on our understanding of how a scarcity mindset may influence social emotions and behaviors.

## Introduction

As a perennial global problem, resource scarcity has impacted humanity throughout its history (Zhao and Tomm, 2018). Unfortunately, many resources have become much more unobtainable during the global coronavirus disease 2019 pandemic (Sachdeva *et al.*, 2021). A scarcity mindset emerges when people perceive the shortage of a resource (Shah *et al.*, 2012). People in a scarcity mindset tend to experience negative emotions such as stress and lack of confidence (Haushofer and Fehr, 2014; Huijsmans *et al.*, 2019) and shift their attention to short-term needs (Shah *et al.*, 2012, 2015). Previous studies suggest that this mindset has adverse effects on cognitive functions, such as attention, executive control and memory (Mani *et al.*, 2013; Mullainathan and Shafir, 2013; Tomm and Zhao, 2017; Zhao and Tomm, 2017; Mani et al., 2020) and can dampen the decision-making process (Cook and Sadeghein, 2018; Huijsmans *et al.*, 2019; Norris *et al.*, 2019; Wiedmer *et al.*, 2020).

Importantly, scarcity mindsets can also induce social problems. For instance, in such a mindset, people might loosen their moral standards (Sharma *et al.*, 2014) or tend to violate social norms (Chang *et al.*, 2022). The incidence of antisocial behavior has also been reported to increase with the degree to which people perceive a scarcity of resources (Prediger *et al.*, 2014). Furthermore, studies have shown that a scarcity mindset activates a strong self-centered bias in social decision-making, which makes individuals less prosocial in their sharing behavior (Roux *et al.*, 2015; Cui *et al.*, 2022).

Empathy is a basic psychological motivator and the key to understanding prosocial behaviors (Singer and Lamm, 2009; Zaki and Ochsner, 2012; De Waal and Preston, 2017; Stevens and Taber, 2021). The literature recognizes that empathy can be categorized into affective and cognitive components (Decety and Lamm, 2006). The affective component describes early emotional contagion and affective sharing, while the cognitive component refers to late cognitive control that is modulated by attention to stimuli (Decety and Lamm, 2006; Gu and Han, 2007; Fan and Han, 2008). Empathy allows us to resonate with the affective states of others and understand their feelings and thoughts (Decety and Jackson, 2004; Singer, 2006; Hein and Singer, 2008). When observing people suffering, individuals can resonate with the painful feelings, which triggers a specific empathic response (i.e. empathy for pain; Singer and Klimecki, 2014). Empathizing with others is crucial for normal social interactions (Fan and Han, 2008; Hetu *et al.*, 2012), and examining how scarcity mindsets affect empathy is essential for understanding and predicting the influence that scarcity mindsets have on social behavior.

To the best of our knowledge, no study has examined whether or not scarcity mindsets influence empathy. Without this knowledge, explaining or predicting social behavior and interpersonal interactions when resources are scarce is very difficult. However, resource scarcity can be considered a source of stress, and studies indicate that stress can reduce empathy. For example, stress induced by the Trier Social Stress Test (TSST) made individuals indifferent, indicating decreased empathic responses to others’ pain (Buruck *et al.*, 2014). In particular, males exhibited enhanced emotional self-centered bias and did not empathize with the feelings of others under TSST-induced stress (Tomova *et al.*, 2014). Additionally, patients with post-traumatic stress disorder display less affective empathy than do healthy individuals (Mazza *et al.*, 2015; Palgi *et al.*, 2017). On the flipside, reducing stress resulted in the emotional contagion of pain in human strangers (Martin *et al.*, 2015). Taken together, we can conclude that stress suppresses empathic responses when observing pain in others.

Studies of event-related potentials (ERPs) have shown that empathizing with others’ pain enhances several electroencephalography (EEG) components. First, it enhances the fronto-central N1 and N2 components, which reflect an early and automatic bottom-up process. Second, it enhances the centro-parietal P3 and late positive potential (LPP) components, which reflect a late cognitive top-down process (Fan and Han, 2008; Cui *et al.*, 2016a, 2017; Coll, 2018; Peng *et al.*, 2019; Meng *et al.*, 2020). Importantly, Gonzalez-Liencres *et al.* (2016) reported reduced empathic ERP responses under TSST-induced stress, as evidenced by the significant difference in P3 amplitudes for unstressed participants when observing others receiving either painful or nonpainful stimuli, but the comparable P3 amplitudes for stressed participants when viewing the same sets of painful or nonpainful stimuli.

The present study thus examined how an induced scarcity mindset modulates empathy for others’ pain. In the literature, budget assignments (Shah *et al.*, 2012), reminder of scarcity cues (Griskevicius *et al.*, 2013; Mani *et al.*, 2013; Li *et al.*, 2021), episodic recall tasks (Roux *et al.*, 2015; Mehta and Zhu, 2016), scenario-based role-playing (Wiedmer *et al.*, 2020) and scarcity word priming (Krosch and Amodio, 2014) have all been used to induce scarcity mindsets. Most of these approaches induce the scarcity mindset before the main task and cannot control how long it lasts. Here, we used the stage-game paradigm created by Huijsmans *et al.* (2019), which can systematically manipulate two different mindsets, which are not influenced by the individual’s life experiences. This paradigm manipulates the number of tokens for simple cognitive/perceptual games, which are unrelated to the main task. The mindset manipulation was divided into three stages, with the main task interspersed between each stage. Thus, a sustained mindset could be induced throughout the experiment.

We compared the differences in behavioral and neural responses between mindsets when viewing either painful or nonpainful pictures. Based on the literature mentioned previously, we hypothesized that at the behavioral level, empathic responses to others’ pain would be smaller for individuals in a scarcity mindset than for those in an abundance mindset. At the neural level, based on previous results (Gonzalez-Liencres *et al.*, 2016), we expected the others’ pain/non-pain differential in late ERP component (P3 or LPP) amplitudes to be smaller when in a scarcity mindset than when in an abundance mindset.

## Materials and methods

### Participants

We recruited 60 right-handed healthy participants as paid volunteers. All had normal or corrected-to-normal vision, and none had neurological disorders, brain injury or developmental disabilities. The study was approved by the Medical Ethical Committee of Shenzhen University in accordance with all provisions of the Declaration of Helsinki. All participants provided written informed consent. One participant was excluded due to her suspicion about the experimental manipulation. Data from 59 participants were thus included in the analysis (see Table 1).

**Table 1.** Demographic and psychometric variables for two groups of participants (mean ± SD)

	Scarcity group	Abundance group	Statistics (*P*)
*Demographic variables*			
Age (years)	21.90 ± 2.23	21.07 ± 2.43	0.178
Female/male	13/16	15/15	–
*Psychometric variables*			
Anxiety (STAI)			
State anxiety	36.34 ± 7.59	39.17 ± 7.81	0.165
Trait anxiety	44.21 ± 9.08	47.10 ± 7.76	0.193
Empathy (QCAE)	86.93 ± 7.89	87.43 ± 7.68	0.805

*Note*: Statistics were obtained using independent-sample *t*-tests.

### Stimuli

The stimuli used in the experiment were pictures showing a person’s body parts in painful or nonpainful situations (termed painful or nonpainful images). We selected 72 pictures for which pain intensity, emotional valence and arousal level had been evaluated via 9-point Likert scales in a previous ERP study (Meng *et al.*, 2013). Significant differences were observed between painful and nonpainful pictures on all three measures (pain intensity: 5.94 ± 0.67 vs 2.03 ± 0.39, *P* < 0.001; emotional valence: 3.17 ± 0.83 vs 5.21 ± 1.37, *P *< 0.001; arousal level: 5.26 ± 0.53 vs 3.32 ± 0.44, *P* < 0.001). Each painful/nonpainful stimulus subtended a visual angle of 6.94° × 5.22° (width × height).

### Procedure

The study had a 2 × 2 mixed experimental design. The between-participant factor was Mindset (scarcity or abundance), which was manipulated using the stage-game paradigm (Huijsmans *et al.*, 2019). The within-participant factor was Picture (painful or nonpainful). Participants were randomly assigned to the scarcity group or the abundance group.

**Fig. 1.**
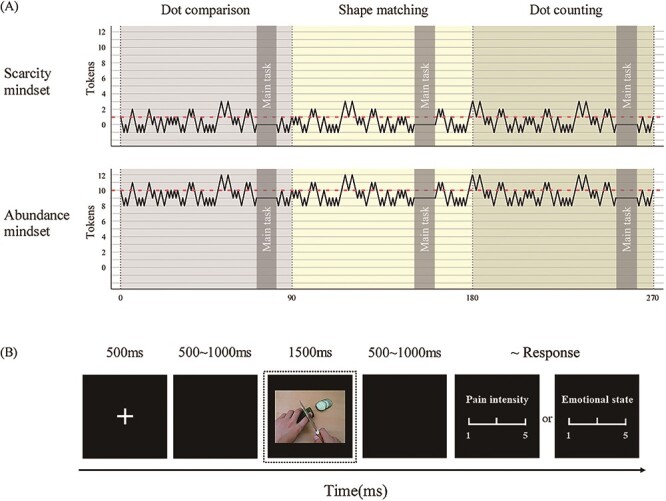
**The experimental design.** (A) The experimental structure. The dotted lines represent the threshold, and the broken lines represent the variation in the number of tokens around the threshold for each stage game in the scarcity (top panel) and abundance (bottom panel) mindsets. The dark shaded sections represent when participants performed the main task. (B) The flowchart describing the main task. ERP responses were locked to the panel of visual stimulus (marked with the dotted box).

**Figure A1.**
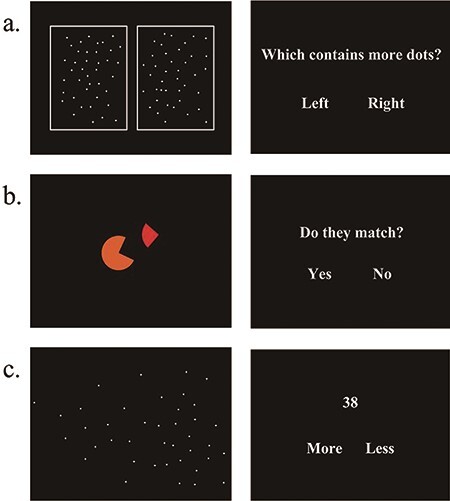
Examples of three-stage games. (i) Dot comparison: participants were asked to choose which box contained more dots. Actually, both boxes always contained an equal number of 30−40 dots. (ii) Shape matching: participants were asked to judge whether the two shapes together could form a perfect circle. Actually, none of the shapes met this criterion. (iii) Dot counting: participants were asked to estimate the number of dots more or less than the presented number (32 or 38). The number of dots was between 30 and 40.

The experiment was conducted in a quiet and temperature-controlled room. The experimental tasks were presented on a 24-inch color monitor using E-prime 3.0 software (Psychology Software Tools Inc., Pittsburgh, PA, USA). The monitor was placed 80 cm from the participants. For the stage games (Huijsmans *et al.*, 2019), each stage was a block, comprising 90 trials of one of the cognitive/perceptual games (dot comparison, shape matching or dot counting; see Appendix A, Figure A1). The number of initial tokens was manipulated, namely, each participant in the scarcity group received one token, while each participant in the abundance group received 10 tokens at the beginning of the task. In each trial, stimuli were presented for 1000 ms, and participants were then requested to answer a question by pressing the ‘F’ or ‘J’ keys during a 2000 ms interval. After the choice was made, feedback (win or lose a token) was presented for 500 ms. Unknown to the participants, the feedback was not related to their choices. It was actually controlled to ensure an equal number of wins and losses within each stage and within each mindset. Specifically, in the scarcity group, the number of current tokens was controlled so that it consistently hovered around the one-token threshold to induce a scarcity mindset (ranged from −1 to 3); in the abundance group, the number of current tokens was controlled to always hover around the 10-token threshold to induce an abundance mindset (ranged from 8 to 12; see Figure 1A). Participants were informed how many tokens they had upon completing each stage game and were required to possess at least one token to progress to the next stage. All participants were clearly informed that if they successfully completed all three-stage games (i.e. they held at least one token at the end of the last game), they would receive a large additional monetary bonus of ￥100. Otherwise, they would only get ￥10 for time compensation. The equal win/loss control ensured that everyone always had at least one token in the end and that all participants would thus finish all games.

Between each stage game, participants were also required to perform the main task. For this task, in each trial (see Figure 1B), a fixation cross was presented for 500 ms on a black screen, followed by a blank interval that varied randomly between 500 and 1000 ms. Then, one of the 72 painful/nonpainful pictures was presented in the center of the screen for 1500 ms, and participants viewed it as instructed. After a blank interval that again randomly varied between 500 and 1000 ms, a 5-point Likert scale was presented, and participants were instructed to respond as accurately as possible with a key-press (from 1 to 5) to judge the pain intensity (1 = no sensation and 5 = unbearable pain) or emotional state (1 = extremely pleasant and 5 = extremely unpleasant) experienced by the person in the picture. The picture disappeared from the screen as soon as the participants responded, indicating the end of the trial. The inter-trial interval was 500 ms. The task comprised 3 blocks of 48 trials (total: 144 trials), which were presented in a pseudo-random order in which each picture was presented twice, once for each type of rating (pain intensity or emotional state). The two presentations of a picture occur in different blocks. Before the formal experiment, participants completed a training session to get acquainted with the procedures.

To assess and control the level of anxiety and empathy in the two groups, participants were instructed to complete the State-Trait Anxiety Inventory (STAI) and the Questionnaire of Cognitive and Affective Empathy (QCAE; Reniers *et al.*, 2011) before the experiment.

To investigate whether participants in the different mindsets experienced different feelings, all participants performed several subjective rating tasks (see Appendix B) related to their stress (1 = extremely stress-free and 9 = extremely stressful), confidence (1 = extremely unconfident and 9 = extremely confident), motivation (1 = extremely unmotivated and 9 = extremely motivated) and excitement (1 = extremely unexcited and 9 = extremely excited) levels. All ratings were performed on 9-point Likert scales four times throughout the experiment: once before the experiment (baseline) and again after each of the three games (post-test: T1, T2 and T3).

### EEG acquisition and analysis

EEG data were recorded from 64 scalp sites using tin electrodes mounted on an actiCHamp system (Brain Vision LLC, Morrisville, NC, USA; passband: 0.01–100 Hz; sampling rate: 1000 Hz). The FCz electrode was used as a recording reference and that on the medial frontal aspect was used as a ground electrode. All electrode impedances remained <5 kΩ.

EEG data were offline pre-processed and analyzed via MATLAB R2019 (MathWorks, Natick, MA, USA) and the EEGLAB toolbox (Delorme and Makeig, 2004). Continuous EEG signals were band-pass filtered 0.1–30 Hz and segmented using a 1200 ms time window (200 ms pre-stimulus and 1000 ms post-stimulus). EEG epochs were baseline-corrected by a 200 ms time interval prior to stimuli onset. Epochs with amplitude values exceeding ±70 μV at any electrode were excluded from the average. EEG epochs were also visually inspected, and trials containing significant noise from gross movements were removed. Electrooculographic artifacts were corrected via the independent component analysis (ICA) algorithm (Jung *et al.*, 2001). These epochs constituted 2.47 ± 2.90% of the total number of epochs. After ICA and an additional baseline correction, EEG trials were re-referenced to the bilateral mastoid electrodes.

For each participant, single-trial ERP waveforms elicited by painful and nonpainful pictorial stimuli were averaged and time-locked to the onset of the stimuli for each mindset condition, thus yielding four average waveforms. Single-participant average ERP waveforms were averaged to obtain group-level ERP waveforms, and group-level scalp topographies at corresponding peak latencies were computed by spline interpolation. According to the topographical distribution of the grand-averaged ERPs and the literature (Li *et al.*, 2020; Wu *et al.*, 2020), the dominant ERP components were as follows: N1 (F1, Fz, F2, FC1, FCz and FC2) within the latency interval of 125–145 ms, N2 (F1, Fz, F2, FC1, FCz and FC2) within the latency interval of 235–255 ms, P3 (CP1, CPz, CP2, P1, Pz and P2) within the latency interval of 355–375 ms and LPP (C1, Cz, C2, CP1, CPz and CP2) within the latency interval of 450–800 ms.

### Statistical analysis

Statistical analysis was conducted using IBM SPSS Statistics 22 (IBM Corp., Armonk, NY, USA). Behavioral data (pain intensity and emotional state ratings) and ERP data were analyzed using two-way repeated-measure analyses of variance (ANOVAs), with the between-participant factor of Mindset (scarcity or abundance) and the within-participant factor of Picture (painful or nonpainful). Differential subjective ratings (post-tests minus baseline) were analyzed using two-way repeated-measure ANOVAs with Mindset (scarcity or abundance) and Time (T1, T2 and T3). If any interaction effect was significant, post-hoc comparisons were performed. The degrees of freedom for *F*-ratios were corrected according to the Greenhouse–Geisser method. Statistical differences were considered significant at *P* < 0.05, and post-hoc *P*-values were Bonferroni-corrected for multiple comparisons. In addition, psychometric scores (STAI-S, STAI-T and QCAE) were analyzed using an independent-sample *t*-test.

## Results

All descriptive results for behavioral and ERP data are expressed as mean ± SE.

### Behavioral data

#### Subjective ratings

**Fig. 2.**
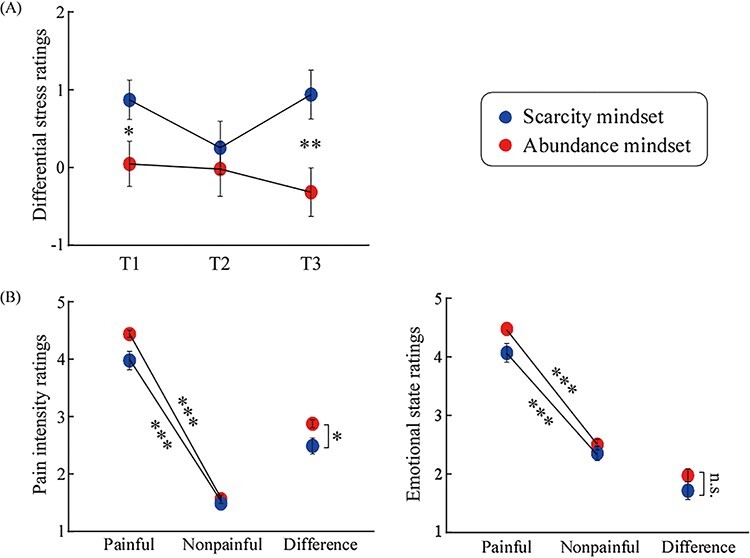
**The behavioral results.** (A) The differential ratings of subjective stress when in the scarcity mindset or the abundance mindset . (B) The ratings of pain intensity (left) and emotional states (right) to painful and nonpainful pictures when in the scarcity mindset or the abundance mindset . Data are expressed as mean ± SEM. *P < 0.05; **P < 0.01; ***P < 0.001; n.s.: *P* > 0.05.

A significant main effect of Mindset on subjective stress ratings [*F*_(1,57)_ = 4.19, *P* = 0.045, η_p_^2^ = 0.07] was observed, such that differential stress ratings were higher in the scarcity mindset than in the abundance mindset (0.71 ± 0.28 vs −0.08 ± 0.27). A significant Mindset × Time interaction [*F*_(2,56)_ = 3.80, *P* = 0.028, η_p_^2^ = 0.12] was also observed. Post-hoc comparisons revealed that differential stress ratings at T1 and T3 were significantly higher in the scarcity mindset than in the abundance mindset (T1: 0.89 ± 0.28 vs 0.07 ± 0.27, *P* = 0.037; T3: 0.96 ± 0.32 vs −0.30 ± 0.31, *P* = 0.006), whereas ratings at T2 were comparable (scarcity: 0.29 ± 0.35; abundance: 0.00 ± 0.35, *P* = 0.566). Differences in confidence, motivation or excitement were not observed between the scarcity and abundance mindsets (see Figure 2A).

#### Pain intensity

A significant main effect of Mindset [*F*_(1,57)_ = 4.69, *P* = 0.035, η_p_^2^ = 0.08] was also observed, such that ratings were significantly lower in the scarcity mindset than in the abundance mindset (2.83 ± 0.06 vs 3.00 ± 0.06). A significant main effect of Picture [*F*_(1,57)_ = 1675.81, *P* < 0.001, η_p_^2^ = 0.97] was observed, such that ratings were significantly higher for painful pictures than for nonpainful pictures (4.28 ± 0.05 vs 1.55 ± 0.05). Critically, a significant Picture × Mindset interaction [*F*_(1,57)_ = 5.05, *P* = 0.028, η_p_^2^ = 0.08] was observed. Post-hoc comparisons revealed that while pain intensity ratings were significantly higher for painful pictures than for nonpainful pictures in both mindsets (scarcity: 4.11 ± 0.07 vs 1.54 ± 0.08, *P* < 0.001; abundance: 4.44 ± 0.07 vs 1.56 ± 0.07, *P* < 0.001), the differential (painful–nonpainful) was significantly lower in the scarcity mindset than in the abundance mindset (2.58 ± 0.62 vs 2.88 ± 0.38, *P* = 0.028) (see Figure 2B). The interaction remained significant even after adding the average stress rating (across the three stages) to the model as a covariate (*P* = 0.009).

#### Emotional state

A significant main effect of Mindset [*F*_(1,57)_ = 4.37, *P* = 0.041, η_p_^2^ = 0.07] was observed, such that ratings were significantly less unpleasant when viewed in the scarcity mindset than when viewed in the abundance mindset (3.32 ± 0.06 vs 3.49 ± 0.06). A significant main effect of Picture [*F*_(1,57)_ = 426.35, *P* < 0.001, η_p_^2^ = 0.88] was observed, such that ratings were significantly more unpleasant for painful pictures than for nonpainful pictures (4.34 ± 0.06 vs 2.47 ± 0.07).

### ERP data

#### N1

A significant Picture × Mindset interaction [*F*_(1,57)_ = 5.75, *P* = 0.020, η_p_^2^ = 0.09] was observed. Post-hoc comparisons revealed that when participants were in the abundance mindset, nonpainful pictures elicited significantly greater N1 amplitudes than did painful pictures (−4.43 ± 0.65 μV vs −3.60 ± 0.63 μV, *P* = 0.006). In contrast, N1 amplitudes did not differ significantly when participants were in the scarcity mindset (nonpainful: −4.83 ± 0.66 μV; painful: −5.00 ± 0.64 μV, *P* = 0.575). The interaction for N1 remained statistically significant after adding the average stress rating to the model as a covariate (*P* = 0.044).

#### N2

A significant main effect of Mindset [*F*_(1,57)_ = 5.53, *P* = 0.022, η_p_^2^ = 0.09] was observed, such that greater N2 amplitudes were elicited in the scarcity mindset than in the abundance mindset (−7.73 ± 0.81 μV vs −5.05 ± 0.80 μV).

#### P3

A significant main effect of Picture [*F*_(1,57)_ = 11.87, *P* = 0.001, η_p_^2^ = 0.17] was observed, such that P3 amplitudes were greater for painful pictures than for nonpainful pictures (5.88 ± 0.63 μV vs 5.03 ± 0.58 μV).

#### LPP

**Fig. 3.**
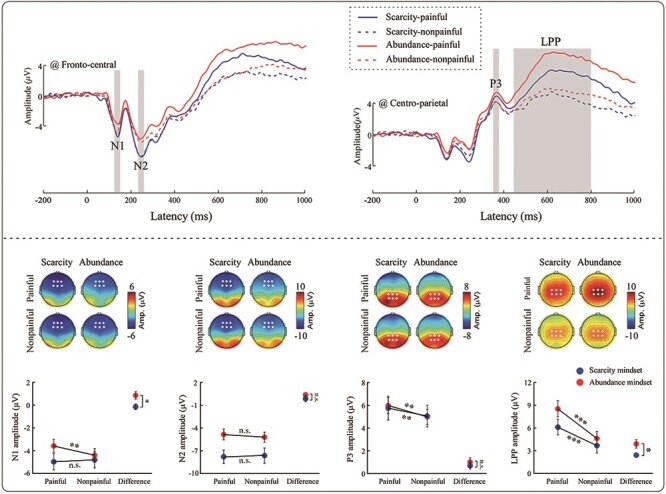
The **ERP results.** Averaged ERP waveforms (top panel) and scalp maps and bar charts (bottom panel) elicited by observing painful (solid) or nonpainful (dotted) pictures in the scarcity or abundance mindsets. Significant differences in ERP waveforms are marked with shaded squares. Electrodes used to estimate ERP amplitudes are marked with points on their respective scalp maps. Data in the bar charts are expressed as mean ± SEM. *P < 0.05; **P < 0.01; ***P < 0.001; n.s.: *P* > 0.05.

A significant main effect of Picture [*F*_(1,57)_ = 96.05, *P* < 0.001, η_p_^2^ = 0.63] was observed, such that LPP amplitudes were greater for painful pictures than for nonpainful pictures (7.32 ± 0.74 μV vs 4.15 ± 0.67 μV). A significant Picture × Mindset interaction [*F*_(1,57)_ = 5.21, *P* = 0.026, η_p_^2^ = 0.08] was also observed. Post-hoc comparisons revealed that while LPP amplitudes were significantly greater for painful pictures than for nonpainful pictures in both mindsets (scarcity: 6.11 ± 1.05 μV vs 3.68 ± 0.95 μV, *P* < 0.001; abundance: 8.53 ± 1.04 μV vs 4.62 ± 0.93 μV, *P* < 0.001), the differential was significantly smaller in the scarcity mindset than in the abundance mindset (2.43 ± 0.31 μV vs 3.91 ± 0.56 μV, *P* = 0.026) (see Figure 3). The interaction effect for LPP remained statistically significant after adding the average stress rating to the model as a covariate (*P* = 0.031).

Furthermore, to test whether responses differed as a function of time during the experiment, behavioral (pain intensity) and ERP (N1 and LPP) data were analyzed using a 2 (Mindset) × 2 (Picture) × 3 (Time: T1, T2 and T3) three-way ANOVAs. Results demonstrated that the effects of the scarcity mindset on empathic responses did not significantly change over time (see Appendix C).

## Discussion

The present study investigated how a scarcity mindset modulates empathy for pain. Behaviorally, compared with the abundance group, the scarcity group reported significantly lower subjective ratings of others’ pain intensity. At the neural level, neural responses to others’ pain (ERP amplitudes) were lower in the scarcity group than in the abundance group, in both early affective stages (N1) and late cognitive stages (LPP) of empathy processing. These results support our hypothesis that empathy for pain is suppressed when in a scarcity mindset.

Huijsmans *et al.* (2019) reported that being less confident and more stressed after task manipulation is evidence of the successful induction of a scarcity mindset. Accordingly, comparisons of subjective stress ratings between the two groups in the current study supported the idea that a scarcity mindset increases feelings of stress. We did not observe a difference in confidence ratings between groups, likely because of the between-subject design. Given the individual differences and the desire to generate persistent mindset manipulation, we assessed subjective ratings at an initial baseline and three times throughout the experiment. After mindset manipulation, the scarcity group reported greater stress than the abundance group after both the first- and last-stage games. During the second-stage game, participants in both groups reported similar stress levels, which were lower than during the other stages. This could be because they gradually adapted to the game setting after the first stage.

Empathy for pain was assessed via pain intensity and emotional state ratings of pictures depicting others in pain (Decety *et al.*, 2010; Kopiś-Posiej *et al.*, 2021). Results showed that when in the scarcity mindset, individuals felt significantly less empathy for pain (painful–nonpainful ratings) than when in the abundance mindset, suggesting that scarcity mindsets induce a diminished empathic response when witnessing others in pain. This difference is evidence of a link between scarcity mindsets and the effects of pain empathy on behavioral reactions. These findings are similar to those of another study: individuals under acute stress supplied significantly lower pain ratings for painful pictures than did those who were not undergoing stress (Buruck *et al.*, 2014). No difference was observed, in the present study, between the two groups in their ratings of others’ emotional states, which was in line with the results by Wu *et al.* (2020). This suggests that the scarcity mindset weakens the cognitive evaluation process involved in empathy for others’ pain.

Additionally, we found that the processing of empathic pain in the brain differed when in the scarcity mindset, at both early and late temporal stages. First, for individuals in the abundance mindset, N1 amplitudes for nonpainful stimuli were higher than those for painful stimuli. In contrast, individuals in the scarcity mindset exhibited similar N1 amplitudes regardless of the type of picture. Our data also showed a positive shift in N1 amplitudes induced by painful stimuli, which was in line with previous research (Fan and Han, 2008; Decety *et al.*, 2010; Wu *et al.*, 2020). Furthermore, given that the N1 component has been suggested to indicate early affective processing of empathic pain responses (Decety *et al.*, 2010; Cui *et al.*, 2016c; Cheng *et al.*, 2017; Meng *et al.*, 2019), relative to individuals who did not perceive resource scarcity, individuals in the scarcity mindset seem unable to discriminate between painful and nonpainful stimuli during the early affective stage. Specifically, early fronto-central modulation of N1 implies automatic processes related to the affective sharing of empathy for pain (Fan and Han, 2008). The effect of scarcity mindset on frontal N1 responses indicated that perceiving insufficient resources inhibited this automatic sharing of others’ feelings of pain. Additionally, because the N1 component is reported to be involved in selective attention (for a review, see Luck *et al.*, 2000), this early effect might have resulted because the scarcity mindset shifted attention orientation from the outset. In brief, the empathic modulation of the N1 component indicates that the suppression of empathy for pain in a scarcity mindset begins at the early automatic, affective processing stage.

Second, while both groups exhibited greater LPP amplitudes for painful pictures than for nonpainful ones, the magnitude of the differential was smaller in the scarcity mindset than in the abundance mindset. A pain effect in the LPP component has been consistently reported when watching others in physical pain (Wu *et al.*, 2020; Liao *et al.*, 2021; Zhuo *et al.*, 2022), and this effect has been suggested to index the late cognitive appraisal of painful stimuli (Cheng *et al.*, 2014). Relative to nonpainful stimuli, increased LPP amplitudes in response to painful stimuli indicate that painful stimuli triggered a more fine-grained evaluation. Furthermore, based on the functional significance of the LPP component, cognitive appraisal of others’ pain was lower for individuals in the scarcity mindset than for those in the abundance mindset. The LPP component over the parietal region is also a neurocognitive indicator of top-down emotion regulation (Hajcak *et al.*, 2010; Myruski *et al.*, 2019; Hajcak and Foti, 2020). Hence, we interpret the effect of the scarcity mindset on LPP responses as evidence of diminished regulation of empathic responses to pain. The Scarcity Mindset Theory proposed that a scarcity mindset causes attentional focus on own current scarce resources and attentional neglect of other important things (Mullainathan and Shafir, 2013). This reduction might arise as a result of attentional narrowing caused by perceiving resource scarcity (Tomm and Zhao, 2016). Due to our experimental design in which participants had to provide empathic ratings after observing pictures, their evaluation began while they were viewing the stimuli. Combined with the behavioral findings (decreased pain intensity ratings), the empathic LPP results suggest that a scarcity mindset reduces late cognitive evaluation processes that lead to empathy for pain.

Gonzalez-Liencres and colleagues (2016) found that stress only decreased the late temporal stage of empathy for pain (P3 component). This is inconsistent with our current findings in which both early (N1 component) and late (LPP component) processing stages of empathy for pain were reduced in the scarcity mindset. In essence, the scarcity mindset is a complicated mixture of many affective states including stress (Huijsmans *et al.*, 2019), which is different from the TSST-induced acute psychosocial stress. We also found that stress ratings as a covariate did not influence the scarcity mindset effect on empathic responses. Thus, even though the scarcity mindset can be regarded as a stressor, it is not the stress per se induced the observed effects on empathic processes. Hence, a scarcity mindset comprehensively inhibited empathic responses to others’ pain, which is not exactly the same as the inhibiting effect of TSST-induced stress in the previous experiment.

Additionally, in the present study, N2 amplitudes over the fronto-central region induced larger negative deflection in the scarcity mindset than in the abundance mindset, regardless of picture type. N2 is an early component indexing emotional contagion in empathy for pain (Cheng *et al.*, 2014; Cui *et al.*, 2016b). Most probably, more negative-going N2 amplitudes in the scarcity mindset than in the abundance mindset reflect a general decline in the processing of others’ emotion, without distinguishing whether they are in pain or not. On the other hand, N2 amplitude also correlates with inhibition or cognitive control (Jodo and Kayama, 1992; Folstein and Van Petten, 2008; Cragg *et al.*, 2009; Brydges *et al.*, 2014). People who experience scarcity are often assumed to lack inhibition and behave badly (Allen *et al.*, 2016; Bratanova *et al.*, 2016; Kristofferson *et al.*, 2016). However, our unexpected finding that the scarcity group showed a larger N2 response suggests that the reduced empathy in the scarcity mindset might be associated with increased response inhibition. One interpretation of this finding is that people in a scarcity mindset would save limited cognitive resources to settle their immediate issues related to insufficient resources. Thus, automatic focusing on others’ emotions would be inhibited. As other research has indicated (Effron and Miller, 2011), when necessary, people experiencing scarcity might still be capable of inhibition control. Thus, we should not oversimplify the issue by falling back on biases and stereotypes.

Similar to numerous ERP studies (Cui *et al.*, 2016a; Jiao *et al.*, 2017; Wu *et al.*, 2020; Kopiś-Posiej *et al.*, 2021), we also observed a late pain effect. We demonstrated an increase in P3 amplitudes over the centro-parietal region when observing others in pain relative to observing others in nonpainful situations. The P3 component over the parietal region reflects late cognitive processing that is associated with extensive evaluation (Fan and Han, 2008) and attention allocation (Hajcak *et al.*, 2010) to emotional stimuli. Painful stimuli can grab attention (Fan and Han, 2008); therefore, individuals may allocate more attentional resources to view and evaluate the painful content of visual stimuli.

Despite the previous implications, some limitations should be noted in the present study. First, We did not directly compare scarcity-induced stress with other sources of stress, such as the TSST, so it would be worthwhile to explore whether the effects observed in the present study can be generalized to other forms of induced stress. Second, although a sample size of about 30 participants per group is commonly used for between-participant ERP studies (Li *et al.*, 2020; Lin *et al.*, 2022), given current replication issues in psychology, a larger sample size might help avoid unreliable *P*-values. Therefore, our findings should be verified in future studies that have larger sample sizes. Third, given that it is easy to increase the likelihood of bogus effects when conducting multiple analyses across different ERP components (Luck and Gaspelin, 2017), it needs replications in the future to demonstrate the robustness of our findings.

In conclusion, our data revealed that empathy for pain was disturbed by a scarcity mindset, not only in the early affective sharing stage of emotional processing, as shown by suppressed N1 responses, but also in the late cognitive evaluation stage, as shown by attenuated LPP responses and pain intensity ratings. We provided empirical evidence regarding the relationship between scarcity mindsets and empathy, which can help us to understand and predict the influence of resource scarcity on human society more comprehensively.

## Data Availability

The data associated with this study can be found at https://pan.baidu.com/s/1KhqScqhDSDv426QHPNwHjw?pwd=2022.

## Appendix B

1. Questions for the main task:

***Pain intensity***. Please use the number keys on the keyboard to rate the intensity of the pain experienced by the person in the picture. Choose your ratings according to the 5-point Likert scale on the screen (1 for no sensation and 5 for unbearable pain).

***Emotional state***. Please use the number keys on the keyboard to rate the intensity of emotions experienced by the person in the picture. Choose your ratings according to the 5-point Likert scale on the screen (1 for extremely pleasant and 5 for extremely unpleasant).

1. Questions for the subjective rating task:

When you got 1 (or 10) token(s) at the beginning of a game, how stressful did you feel?

When you got 1 (or 10) token(s) at the beginning of a game, how confident did you feel?

When you got 1(or 10) token(s) at the beginning of a game, how motivated did you feel?

When you got 1 (or 10) token(s) at the beginning of a game, how excited did you feel?

## Appendix C

To test whether responses differed as a function of time during the experiment, behavioral (pain intensity) and ERP (N1 and LPP) data were analyzed using a 2 (Mindset) × 2 (Picture) × 3 (Time: T1, T2 and T3) three-way ANOVAs. Results were as follows:

***Pain intensity.*** A significant main effect of Time [*F*_(2,56)_ = 5.97, *P* = 0.004, η_p_^2^ = 0.18] was observed, such that ratings were significantly higher at T1 than at T2 (3.00 ± 0.05 vs 2.86 ± 0.05, *P* = 0.003). In contrast, ratings at T1 and T3 (3.00 ± 0.05 vs 2.88 ± 0.04, *P* = 0.056), as well as ratings at T2 and T3 (2.86 ± 0.05 vs 2.88 ± 0.04, *P* = 1.000), did not differ significantly. A significant Time × Mindset interaction [*F*_(2,56)_ = 5.27, *P* = 0.008, η_p_^2^ = 0.16] was observed. Post-hoc comparisons revealed that at T2, ratings were significantly higher in the abundance mindset than in the scarcity mindset (3.01 ± 0.07 vs 2.72 ± 0.07, *P* = 0.003); at T3, a similar but weaker trend was observed (2.96 ± 0.06 vs 2.81 ± 0.06, *P* = 0.091); at T1, the trend was the same, but the difference was smaller (3.04 ± 0.07 vs 2.96 ± 0.07, *P* = 0.390). A significant Time × Picture interaction [*F*_(2,56)_ = 15.45, *P* < 0.001, η_p_^2^ = 0.36] was also observed. Post-hoc comparisons revealed that while ratings were significantly higher for painful pictures than for nonpainful pictures at all time points (*Ps* < 0.001), the differential (painful–nonpainful) was higher at T3 than at T2 (2.88 ± 0.07 vs 2.59 ± 0.08, *P* < 0.001). In contrast, differential ratings at T1 and T2 (2.72 ± 0.09 vs 2.59 ± 0.08, *P* = 0.377), as well as those at T1 and T3 (2.72 ± 0.09 vs 2.88 ± 0.07, *P* = 0.175), did not differ significantly. The three-way interaction was not significant [*F*_(2,56)_ = 1.49, *P* = 0.234, η_p_^2^ = 0.05].

***N1.*** A significant main effect of Time [*F*_(2,56)_ = 7.31, *P* = 0.001, η_p_^2^ = 0.11] was observed, such that pictures elicited significantly greater N1 amplitudes at T2 than at T1 (−5.03 ± 0.46 μV vs −4.03 ± 0.44 μV, *P* < 0.001) or T3 (−5.03 ± 0.46 μV vs −4.33 ± 0.51 μV, *P* = 0.048), whereas N1 amplitudes at T1 and T3 (−4.03 ± 0.44 μV vs −4.33 ± 0.51 μV, *P* = 0.880) did not differ significantly. The three-way interaction was not significant [*F*_(2,56)_ = 1.64, *P* = 0.204, η_p_^2^ = 0.06].

***LPP.*** A significant main effect of Time [*F*_(2,56)_ = 3.57, *P* = 0.035, η_p_^2^ = 0.11] was observed such that pictures elicited significantly greater LPP amplitudes at T1 than at T2 (6.39 ± 0.73 μV vs 5.50 ± 0.69 μV, *P* = 0.33). In contrast, LPP amplitudes at T1 and T3 (6.39 ± 0.73 μV vs 5.82 ± 0.80 μV, *P* = 0.560), as well as those at T2 and T3 (5.50 ± 0.69 μV vs 5.82 ± 0.80 μV, *P* = 1.000), did not differ significantly. The three-way interaction was not significant [*F*_(2,56)_ = 1.19, *P* = 0.311, η_p_^2^ = 0.04].
